# The Rivas Cohort Study: design and baseline characteristics of a Nicaraguan cohort

**DOI:** 10.1186/s12882-016-0320-9

**Published:** 2016-07-25

**Authors:** Kailey Minnings, Madeline Fiore, Martha Mosco, Ryan Ferguson, Sarah Leatherman, Eric Kerns, James Kaufman, Melissa Fiore, Daniel Brooks, Juan Jose Amador, Hillary Paulsen, Zachary Ernstberger, Bricia Trejo, Elyse Sullivan, Amos Lichtman, Keriann Nobil, Matthew Lawlor, Cassandra Parker, Rulan Parekh, Louis Fiore

**Affiliations:** University of Toronto Faculty of Medicine, 1 King’s College Circle, Toronto, ON M5S 1A8 Canada; UMass Medical School, 55 N Lake Ave, Chestnut St. Apt 21, Worcester, MA 01604 34 UK; UMass Medical School, 55 N Lake Ave, Chestnut St. Apt 21, Worcester, MA 01609 UK; Beth Israel Deaconess Medical Center, 330 Brookline Ave, Boston, MA 02215 USA; Beth Israel Deaconess Medical Center, 16 Oak Manor Dr. Barrington, Barrington, RI 02806 USA; Massachusetts Veterans Epidemiology Research and Information Center Health, 150 South Huntington Avenue, Boston, MA 02130 USA; VA Boston Healthcare System, 150 South Huntington Ave, Boston, MA 02130 USA; Warren Alpert Medical School of Brown University, 593 Eddy Street, APC 9, Providence, RI 02903 USA; VA New York Healthcare System, 423 East 23rd Street, New York, NY 10010 USA; Boston University School of Public Health, 715 Albany Street, T317E, Boston, MA 02118 USA; Boston University, 715 Albany St., Talbot 3 East, Boston, MA 02118 USA; Athena Health, 311 Arsenal Street, Watertown, MA 02472 USA; Athena Health, 18 Parker Street, Cambridge, MA 02138 USA; Boston Children’s Hospital, 401 Park Dr., 6th Floor, Landmark Center, Boston, MA 02115 USA; Boston Children’s Hospital, 12 Greenway Ct. Apt. 3, Brookline, MA 02446 USA; Indiana University School of Medicine, 340 West 10th Street, Suite 6200, Indianapolis, IN 46202 USA; Indiana University School of Medicine, 1547 N. College Ave, Indianapolis, IN 46202 USA; Boston University School of Public Health, 801 Massachusetts Avenue 4th Floor, Boston, MA 02118 USA; Boston University School of Public Health, 60 Parkway Road, Newton, MA 02460 USA; Northeastern University, 350 Huntington Ave, Boston, MA 02115 USA; Northeastern University, 255 Newbury Street, Apr1R, Boston, MA 02116 USA; UMass Medical School, 55 N Lake Ave, Worcester, MA 01604 USA; UMass Medical School, 60 Parkway Rd., Newton, MA 02460 USA; University of Massachusetts Medical School, 55 N. Lake Ave, Worcester, MA 01605 USA; University of Massachusetts Medical School, 285 Plantation St Apt 522, Worcester, MA 01604 USA; Boston University Schools of Medicine and Public Health, 72 E. Concord Street, Boston, MA 02118 USA; Boston University Schools of Medicine and Public Health, 255 Newbury St 1R, Boston, MA 02116 USA; Boston University Schools of Medicine and Public Health, 160 E Berkeley St. Apt 304, Boston, MA 02118 USA; Hospital for Sick Children, University Health Network and University of Toronto, 555 University Ave, Toronto, ON M5G1X8 Canada

**Keywords:** Cohort studies, Nicaragua, Central America, Renal insufficiency, Public health, Public health surveillance, Data collection, Baseline Survey, Creatinine

## Abstract

**Background:**

A lack of advanced healthcare information systems and validated scientific cohorts in Nicaragua makes it difficult to estimate disease prevalences and other public health statistics. Although there is concern of an “epidemic” of chronic kidney disease (CKD) in this country, statistics regarding its magnitude are derived from only a small number of non-representative studies. Budgetary constraints and the logistical problems of maintaining a study cohort make longitudinal studies difficult. The Rivas Cohort was created to measure disease burden of CKD and other public health priorities in the Department of Rivas, Nicaragua. Using primarily volunteer research students and technologic innovation including GPS, digital photography and point of care biochemical analysis, the ability to establish a longitudinal chronic disease cohort is demonstrated.

**Methods:**

Subjects were recruited from consecutive adjacent households in thirty-two randomly selected communities in the ten municipalities that comprise the Department of Rivas in southern Pacific coastal Nicaragua. The study was conducted in two phases. In the first phase, subjects were enrolled into the cohort and consented for future re-contact. In Phase II, conducted two years later, attempts were made to re-contact 400 of these subjects for additional data collection. Demographic, lifestyle, occupational, exposure and health data was collected for both phases of the study. Blood and urine testing and height, weight and blood pressure measurements were also performed. GPS coordinates of homes were recorded and maps of remote communities created.

**Results:**

Of 1397 adults living in 533 households approached for participation a total of 1242 (89 %) were enrolled in the cohort. The median age is 41 years and 43 % are male, demographics in agreement with Nicaraguan census data for the Department of Rivas. During Phase II we attempted to re-contact 400 subjects for a follow-up study of CKD. It was possible to re-contact 84 % of these participants and of those re-contacted 95 % agreed to participate in the follow-up study. Of subjects that were not successfully re-contacted the majority had either moved (32) or were not at home (22) at the time of the study team visits.

**Conclusion:**

The Rivas Cohort Study enrolled a representative sample of 1242 adults living in the Department of Rivas, Nicaragua. The high re-contact and participation rates at two years suggests that the cohort is suitable for long-term studies and presents opportunities for investigations of disease prevalence, incidence, treatment and other public health matters. GPS coordinates and maps are available for future researchers who wish to use the cohort for additional studies.

**Electronic supplementary material:**

The online version of this article (doi:10.1186/s12882-016-0320-9) contains supplementary material, which is available to authorized users.

## Background

### Introduction

The lack of reliable healthcare system data in some developing countries makes it difficult to estimate disease incidence and prevalence and can result in delayed recognition of emerging public health problems [1]. Epidemiological studies, launched after such issues are initially suspected are difficult and expensive to conduct in what are often remote and rural areas with poor healthcare system and transportation infrastructure. These logistical difficulties result in studies with important limitations due to small sample size, bias introduced by subject recruitment methodology and the inability to follow subjects over prolonged intervals of time necessary to estimate disease incidence. Chronic kidney disease of unknown origin (CKDu) has recently been recognized as an ‘epidemic’ in Central America [2–4] and has received much attention in the lay press, but epidemiological studies of the entity suffer from these considerations.

Studies of targeted populations, conducted in northwestern Nicaragua and El Salvador between 2008 and 2014, have reported prevalences of CKDu to be between 10 and 40 % with higher rates seen in populations enriched for male agriculture workers [5–10], miners [8], older individuals, participants living at altitudes less than 500 m above sea level [11–13] and those who consumed lija, a home-brewed alcoholic beverage [9]. While these cross-sectional studies of targeted communities identify potential risk factors for CKDu, the broad range of disease prevalence estimates they generate make it difficult to appreciate the impact of the disease across the general population. A particular difficulty in doing longitudinal studies in rural communities in Nicaragua is re-locating visited homes as they typically do not have addresses and roads are generally unnamed.

This report describes the establishment and stability characteristics of a representative geographical-based cohort to study CKDu and other public health issues in Rivas, the southernmost department (a geographical area with administrative responsibilities that correspond to a county in the United States) in Nicaragua that borders the Pacific Ocean. We studied the Department of Rivas at the request of local public health officials who wished to confirm their impression that prevalence of CKD in their region was as high as it was in Northern Nicaragua where the ‘epidemic’ was first identified. The two Departments share occupational and environmental risk factors for CKD. Rivas has a population of 156,283 and has an area of approximately 2100 km^2^ [14]. Overall, 52.6 % of the inhabitants of the department of Rivas are considered to be living in rural areas, with 52 % of men and 8 % of women working primarily in agriculture [14]. The study was done with minimal cost by using volunteer research associates and longitudinal follow up was enhanced through the use of global positioning system (GPS) technology to precisely identify participants’ residences.

### Research objective

The research objective was to establish a cohort of at least 1000 adults representative of the population of Rivas and to determine the ability to maintain follow up of the cohort over a two year time period. Questionnaire, physical exam and laboratory data collected from participants at cohort inception and over time would be useful to study prevalence, incidence and risk factors for CKDu and other public health problems. To the extent that Rivas is reflective of the population and environment of other regions of Nicaragua, the data from this cohort could be used to understand similar public health issues in other departments of Nicaragua.

## Methods

The study was conducted in two phases. In Phase I (2012) subjects were recruited into the cohort from communities in the Department. In Phase II (2014) these same communities were revisited and an attempt was made to re-contact 400 of the 1021 subjects enrolled in Phase I. Additional subjects were enrolled in Phase II to increase the size of the cohort.

### Phase I

#### Setting

Phase I was conducted in eight of the ten municipalities that comprise the Department of Rivas; Rivas (pop. 41080, 67 % urban), Tola (pop. 22012, 26.4 % urban) Belen (pop. 16428, 39.3 % urban), San Juan del Sur (pop. 14741, 49.0 % urban), Potosi (pop. 11904, 39.9 % urban), San Jorge (pop.8024, 88 % urban), Buenos Aires (pop. 5420, 41.3 % urban) and Cardenas (pop. 6990, 13 % urban) [14]. Two municipalities, (Moyogalpa and Altagracia on the island of Ometepe) excluded from Phase I due to logistical difficulties of travel were included in Phase II.

Subject recruitment occurred in selected communities within each municipality over the 8 week period from June 15 to August 15, 2012. Communities were randomly selected by overlaying a grid with numbered squares on maps of the municipalities and then using a random number generator to determine which grid-square would be targeted on a given recruitment day. Typically three or four communities per municipality were visited. On two occasions the randomly selected communities could not be used to recruit subjects - one community was privately owned and not accessible and the second was not inhabited. In both cases an abutting community was instead studied. Each of the communities is less than 400 m above sea level.

Field work was conducted by research teams that consisted of two or three research assistants with at least one Spanish-speaking interviewer on each team. The number of teams operating at any time varied depending with the number of research assistants in-country, but was typically at least two teams daily. Team members were generally North American university, medical, nursing and public health students recruited through social networking and word of mouth. Selection criteria included a minimum of basic Spanish speaking skills, an interest in Global Health as a possible career choice, phlebotomy expertise and a strong recommendation by their school faculty. All research assistants were educated about the goals of the project and were trained on interview technique, data recording, specimen handling and data confidentiality by the project principle investigator (LDF). Interested students were referred to a recruitment web page where application forms were located. Research assistants paid for their own travel to Nicaragua and room and board while there. Home stays were arranged for students who chose to live with local families.

The research team was based in the town of San Juan del Sur and travelled on average approximately one and a half hours in four wheel drive SUVs to reach the communities selected for recruitment. Roads from the Pan-American Highway to the communities were unpaved and not well maintained. Poor signage made use of local drivers essential.

#### Subject recruitment and data collection

Once the selected community was reached the research team approached consecutive houses sequentially until a predetermined end-time was reached. Only a small fraction of households in any one community could be approached for participation in this time period. Although subjects were recruited from the majority of households visited, no record was kept of the number of unoccupied homes. The research team presented the project to household members by reciting a standardized introductory script. Enrollment was limited to adults (18 years of age or older) currently living in the selected households. A record was maintained of household members who refused participation. To minimize the impact of work restricting participation, sampling was done during lunch hours and on weekends as much as possible and multiple visits were made to each community to try to enroll complete households.

Willing participants gave written consent to complete an orally administered questionnaire and provide a blood sample for creatinine determination via finger stick and a urine sample for dipstick testing. Content areas of the questionnaire included personal identification and contact information, basic demographics, household membership, health symptoms, hydration practices, occupational and exposure history, and personal and family past medical history (see Additional file 1). Questionnaire responses and biological sample testing results were recorded directly onto forms and later entered into a Microsoft Access database. Contact information was collected for eligible persons who were not home at the time of the visit and at least one additional attempt was made to revisit the community to enroll them.

Participants whose creatinine measurement exceeded 1.5 mg/dL (a value considered to indicate possible chronic kidney disease) using a handheld point of care device were given a record of their results to bring to the local community health center. Abnormal urine dipstick test results were communicated to participants for the following indicators: glucose, erythrocytes and protein. Results were communicated to the participant using carefully developed scripts to avoid confusion or emotional stress.

#### Mapping of participant homes

**Fig. 1:**
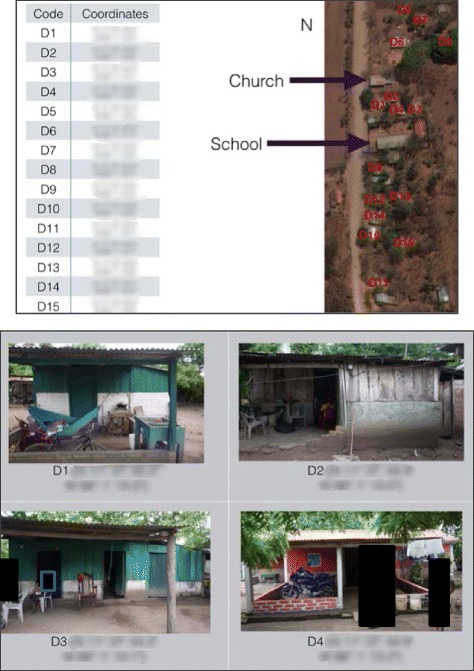
Example of a map with GPS coordinates and photos of homes used to re-locate study subjects

The rural setting of many homes and the lack of addresses or detailed maps made it important to record precise locations of households so that future visits would be possible. Photos and global positioning system (GPS) coordinates of each home (using a Garmin GPS device) were obtained and Google Earth Satellite images of each community with households and important landmarks identified were compiled (Fig. 1). Cellular phone numbers were collected when available.

#### Laboratory analysis

Capillary blood creatinine was determined using the StatSensor Express (Nova Biomedical, Waltham, MA, USA) point-of-care creatinine measuring system with well-defined performance characteristics [15]. Blood samples were obtained by finger stick using Dynarex Sensilance Safety Lancets (21 Gauge). When possible, repeat testing was done immediately using a different finger on individuals whose creatinine value exceeded 1.4 mg/dL. Urine dipstick testing was done immediately on voided specimens using Rapid Response 10 Parameter (10SG) Urinalysis Reagent Test Strips (BTNX INC, Markham, Ontario).

### Phase II

The aims of Phase II were to determine the ability to maintain follow up of the cohort over a two year time period, to gauge whether enrolled subjects were willing to participate in a follow-up study of CKD and to expand the cohort by enrollment of additional subjects.

#### Setting

Two years after cohort inception the research team returned to 29 of the 30 communities initially visited and made first visits to the municipalities of Myogalpa (pop. 9729, 49.3 % urban) and Altagracia (pop. 19955, 35.3 % urban) located on the island of Ometepe. Subject enrollment and revisits occurred over an 8 week period from June 14 to August 15, 2014. All communities with pre-selected cohort participants were revisited at least twice to maximize the number of subjects re-contacted.

#### Subject recruitment and data collection

Four hundred individuals enrolled in Phase I were pre-selected for a follow-up visit based on creatinine testing in 2012 that classified them as ‘potential cases’ of chronic kidney disease (estimated glomerular filtration rate less than 60 ml/min/1.73 m^2^ as calculated by the MDRD equation) [16]. The research team, consisting of 2–3 research assistants with at least one fluent in Spanish and using the maps, GPS information and names collected during Phase I, revisited communities to locate these individuals. Three of 10 research assistants who conducted phase II had participated in phase I of the study. One community was not revisited (Papaturro) as no subjects there were selected for a follow-up visit.

Once identified the potential cases were asked to provide informed consent for Phase II data collection including height, weight, two blood pressure measurements (one from each arm when possible) and a venous blood sample for creatinine determination. Where potential cases were not home or had moved additional arrangements (such as travelling to their new communities) were made to contact them. Photos and GPS coordinates were collected for cohort members who had changed residence.

Attempts were also made to enroll household members who were living in the home at the time of Phase I enrollment but were either not at home or had declined participation at that time. Once identified these subjects were enrolled as new cohort participants following enrollment procedures described in Phase I with the addition of a venous blood draw in lieu of a finger stick for creatinine determination. Basic demographic information (age, sex and occupation) was collected if these household members were not available or refused study enrollment.

Finally, new subjects were recruited into the cohort from households in the Departments of Myogalpa and Altagracia, two municipalities not visited in 2012, using the community and household selection process followed in Phase I.

Participants were informed at the time of informed consent that they would be notified if their creatinine determination exceeded 1.5 mg/dl if male and 1.2 mg/dl if female. These results were communicated using carefully worded scripts and subjects were encouraged to follow-up with their care provider at their local health center.

#### Laboratory analysis

Blood samples were obtained by trained phlebotomists using BD Safety-Lok blood collection sets with 21 and 23 gauge needles. Blood samples were placed on ice immediately after collection, centrifuged at 3000 g for 15 min within 3 h and then refrigerated until transport (once weekly) to the ISO certified Centro Nacional de Diagnostico y Referencia (CNDR) in Managua, a division of the Ministry of Health (MINSA), and stored at −80 C. Serum creatinine was measured at CNDR using a kinetic-rate Jaffe method; calculations were made to calibrate to an isotopic dilution mass spectrometry (IDMS) standard. Urine dipstick testing was performed for newly enrolled participants following the same procedure as described in Phase I.

## Results

### Enrollment

Study enrollment is presented in Table 1. A total of 490 households were visited during Phase I of the study period and an additional 43 were visited in Phase II. Of the 1659 adults living in those homes, 262 were not available for interview at any of the study visits, mostly because they were at work or travelling. A total of 1242 of the 1397 adults approached agreed to participate and were enrolled in the study (1021 during Phase I and 221 during Phase II). The most common reason for refusal was unwillingness to allow a finger stick or blood draw. Demographic information including age, sex and occupation was collected for 252 of the 417 non-enrolled (includes refusals and those not available) household members (Table 2). Participation rates by municipality are given in Table 3.

**Table 1:** Summary of participation and demographics of participants during Phases I and II

Total number of household visited (Phase I + Phase II)	533
Total number of residents (>18 yo)	1659
Number of enrolled residents (%)	1242 (75 %)
Number of non-enrolled residents (%)	417 (25 %)
Number of residents who refused to participate (%)	155 (9.3 %)
Number of residents unable to be contacted (%)	262 (15.8 %)

**Table 2:** Comparison of age, gender and agricultural worker status of enrolled subjects and the 252 (out of 417) non-enrolled subjects for whom data is available

	Enrolled (*n* = 1242)	Not enrolled (*n* = 252)
Age (yrs), mean, median	40.3, 36.4	36.7, 31.0
Male, %	43.3 %	64.3 %
Past or current agricultural worker, %	48.2 %	44.8 %

All differences between enrolled and not enrolled are significant to the *p* < .05 level

**Table 3:** Participation by municipality, Phases I and II. Municipality population and population density (individuals/Km^2^) are derived from the 2005 Census [14]

Municipality	Municipality population	Municipality population density	Subjects enrolled	Subjects Enrolled per 1,000 population	Household residents never approached	Household residents who refused	Participation rate (%)
Tola	22,012	46.2	157	7.13	21	13	82
Belen	16,428	66.7	154	9.37	10	24	82
Buenos Aires	5420	72.1	104	19.18	8	9	86
San Juan del Sur	14,741	35.9	129	8.75	38	24	67
San Jorge	8024	323.2	158	19.69	45	15	72
Potosi	11,904	82.9	161	13.52	34	16	76
Rivas	41,080	146.4	190	4.63	59	31	68
Cardenas	6990	30.8	105	15.02	19	15	76
Altagracia	19,955	94.5	42	2.10	17	7	64
Moyogalpa	9729	147.5	42	4.31	11	1	78
All Municipalities	156,283	72.3	1242	7.94	262	155	75

### Cohort description

Demographics of the study cohort by municipality are presented in Table 4. The mean age was 40.4 years (median = 36.4, range 18 to 100), 43.3 % were male and the predominant occupation was agriculture worker. The average duration of residence was 32 years in Rivas Department and 20 years in the current home.

**Table 4:** Demographics of subjects enrolled in the cohort (Phases I and II)

	Municipality										
	Altagracia (*N* = 42)	Belen (*N* = 151)	Buenos Aires (*N* = 104)	Cardenas (*N* = 114)	Moyogalpa (*N* = 42)	Potosi (*N* = 161)	Rivas (*N* = 182)	San Jorge (*N* = 157)	San Juan del Sur (*N* = 131)	Tola (*N* = 156)	Overall (*N* = 1,240)
Age, n (%)											
< 20	2 (4.9 %)	6 (4.0 %)	5 (4.9 %)	6 (5.3 %)	2 (5.0 %)	11 (6.9 %)	9 (5.0 %)	6 (3.9 %)	7 (5.4 %)	10 (6.5 %)	64 (5.2 %)
20 – 29	10 (24.4 %)	45 (30.2 %)	33 (32.0 %)	33 (29.2 %)	13 (32.5 %)	39 (24.4 %)	45 (24.9 %)	48 (30.8 %)	41 (31.8 %)	39 (25.3 %)	346 (28.2 %)
30 – 39	7 (17.1 %)	36 (24.2 %)	31 (30.1 %)	25 (22.1 %)	9 (22.5 %)	45 (28.1 %)	43 (23.8 %)	46 (29.5 %)	28 (21.7 %)	25 (16.2 %)	295 (24.1 %)
40 – 49	11 (26.8 %)	28 (18.8 %)	11 (10.7 %)	22 (19.5 %)	7 (17.5 %)	23 (14.4 %)	27 (14.9 %)	23 (14.7 %)	16 (12.4 %)	32 (20.8 %)	200 (16.3 %)
50 – 59	6 (14.6 %)	14 (9.4 %)	10 (9.7 %)	13 (11.5 %)	5 (12.5 %)	19 (11.9 %)	29 (16.0 %)	17 (10.9 %)	20 (15.5 %)	13 (8.4 %)	146 (11.9 %)
60 – 69	3 (7.3 %)	11 (7.4 %)	6 (5.8 %)	11 (9.7 %)	2 (5.0 %)	12 (7.5 %)	13 (7.2 %)	9 (5.8 %)	11 (8.5 %)	15 (9.7 %)	93 (7.6 %)
70 – 79	2 (4.9 %)	6 (4.0 %)	6 (5.8 %)	3 (2.7 %)	1 (2.5 %)	8 (5.0 %)	8 (4.4 %)	5 (3.2 %)	3 (2.3 %)	14 (9.1 %)	56 (4.6 %)
≥ 80	0	3 (2.0 %)	1 (1.0 %)	0	1 (2.5 %)	3 (1.9 %)	7 (3.9 %)	2 (1.3 %)	3 (2.3 %)	6 (3.9 %)	26 (2.1 %)
Age (years), mean (SD), median	40.2 (15.5), 41.2	39.8 (16.6), 6.7	38.2 (16.6), 33.3	39.5 (14.7), 37.3	38.7 (16.4), 34.4	40.4 (16.7), 35.8	42.3 (18.0), 38.2	38.4 (15.4), 34.7	39.8 (16.6), 37.1	43.5 (18.9), 40.6	40.3 (16.8), 36.4
Male, n (%)	22 (52.4 %)	73 (48.3 %)	54 (51.9 %)	47 (41.6 %)	18 (42.9 %)	62 (38.5 %)	68 (37.4 %)	65 (41.4 %)	54 (41.2 %)	74 (47.4 %)	537 (43.3 %)
Years in Rivas, median (IQR)	35.0 (21 – 52)	32.5 (23 – 41)	31.0 (24 – 42)	31.0 (20 – 48)	35.0 (24 – 49)	33.0 (23 – 48)	33.0 (22 – 50)	32.0 (22 – 42)	30.0 (22 – 45)	36.0 (23 – 49)	32.0 (22 – 47)
Years in Home, median (IQR)	15.0 (6 – 25)	22.0 (10 – 32)	12.5 (6 – 23)	15.0 (4 – 24)	24.0 (20 – 33)	20.5 (8 – 32)	22.0 (10 – 35)	15.0 (6 – 32)	12.0 (5 – 25)	24.5 (13 – 41)	20.0 (8 – 31)

Table 5 compares demographic data of the cohort to census data for the Department of Rivas and Nicaragua [14]. Selected baseline questionnaire data from subjects enrolled in the cohort (Phases I and II) is shown in Table 6. Laboratory and physical exam data collected in Phases I and II will be reported in a separate publication.

**Table 5:** Comparison of gender and age distribution of cohort to available census data [14]

		Cohort	Rivas	Nicaragua
Population > 18 yo.		1162	84,858	2,627,612
Age				
20–29	Total	29.8 %	33.1 %	36.2 %
	M	13.0 %	16.6 %	17.6 %
	F	16.8 %	16.5 %	18.6 %
30–39	Total	25.4 %	22.6 %	24.1 %
	M	10.2 %	10.9 %	11.4 %
	F	15.2 %	11.7 %	12.7 %
40–49	Total	17.2 %	17.6 %	17.2 %
	M	7.5 %	8.5 %	8.0 %
	F	9.7 %	9.1 %	9.2 %
50–59	Total	12.6 %	11.6 %	10.6 %
	M	5.0 %	5.8 %	5.1 %
	F	7.6 %	5.8 %	5.5 %
60–69	Total	8.0 %	7.2 %	6.3 %
	M	3.8 %	3.5 %	2.9 %
	F	4.2 %	3.7 %	3.4 %
70–79	Total	4.8 %	5.0 %	3.7 %
	M	2.3 %	2.4 %	1.8 %
	F	2.5 %	2.6 %	1.9 %
> 80	Total	2.2 %	3.0 %	1.9 %
	M	1.1 %	1.3 %	0.8 %
	F	1.1 %	1.7 %	1.1 %
Female		56.7 %	49.7 %	50.7 %

**Table 6:** Selected baseline questionnaire data from subjects enrolled in the cohort (Phases I and II)

Variable			Male (*N* = 537)	Female (*N* = 702)
Households with 1+ mobile phones			368 (68.5 %)	493 (70.2 %)
Water source				
	Well		272 (50.8 %)	311 (44.4 %)
	Piping		236 (44.0 %)	337 (48.1 %)
	River		1 (0.2 %)	1 (0.1 %)
	Spring		0	1 (0.1 %)
	Bottled		26 (4.9 %)	48 (6.9 %)
	Other		16 (3.0 %)	16 (2.3 %)
Drinking water treatment				
	No treatment		303 (56.5 %)	427 (61.0 %)
	Chlorine		182 (34.0 %)	196 (28.0 %)
	Filter		35 (6.5 %)	44 (6.3 %)
Alcohol				
	Reports any alcohol intake (beer +/− rum)		186 (35.4 %)	27 (3.9 %)
	Reported rum intake positive		119 (22.7 %)	9 (1.3 %)
	Reported beer intake positive		157 (29.9 %)	25 (3.6 %)
Work History				
	Reported current work in agriculture		219 (41.2 %)	20 (3.0 %)
	Reported ever working in agriculture		361 (67.5 %)	172 (25.4 %)
		Number of years in agriculture	21.0	17.2
	Reported work in fishing		11 (2.1 %)	0
	Reported work in business		16 (3.0 %)	25 (3.7 %)
	Reported working in ranching		18 (3.4 %)	1 (0.2 %)
	Reported work in service		23 (4.3 %)	44 (6.5 %)
	Reported work in other		202 (38.0 %)	421 (62.0 %)
	Reported manual work		374 (70.3 %)	125 (18.4 %)
	Number of hours/day working		7.9	8.0
	Reported self-employed		222 (41.7 %)	98 (14.4 %)
	Reported work applying fertilizer, herbicides, insecticides, or poison		216 (40.6 %)	15 (2.2 %)
	Reported work mixing poisons or herbicides		233 (43.6 %)	17 (2.5 %)
	Age at first job		18.9	14.4
	Reported ever working in sugarcane		168 (31.4 %)	28 (4.1 %)
Medical History				
	HTN		108 (20.2 %)	228 (32.5 %)
	Diabetes		31 (5.8 %)	51 (7.3 %)
	Kidney stones		30 (5.6 %)	30 (4.3 %)
	1st degree relative with HTN		286 (53.4 %)	385 (54.8 %)
	1st degree relative with Kidney stones		145 (27.1 %)	214 (30.5 %)
	Reported use of pain meds for 3+ days in past 3 months		219 (40.8 %)	385 (54.8 %)
	Reported use of antibiotics in past 3 months		167 (31.1 %)	332 (47.3 %)
	Reported lower body, flank, or back pain in past 3 months		340 (63.3 %)	577 (82.2 %)
	Reported dysuria in past 3 months		212 (39.5 %)	371 (52.9 %)
	Reported fever in past 3 months		155 (28.9 %)	247 (35.2 %)
	Reported weakness/fatigue in past 3 months		255 (47.5 %)	455 (64.8 %)
Current smoker			121 (25.6 %)	22 (3.6 %)

### Cohort stability

Phase II required re-contact of four hundred subjects from Phase I for venous blood draw and additional data collection as a component of a CKD sub-study to be reported elsewhere. Using the maps, GPS coordinates and photographs of homes obtained during Phase I it was possible for the study team to find all households. Of the 400 potential cases of CKD identified during Phase I, 336 (84 %) were successfully located during Phase II (Table 7). Reasons for failure to re-contact the 64 subjects included 10 participants who had died, 32 who had moved and were not found, and 22 people who were not home at any of the multiple study visits and could not be contacted. A total of 319 (95 %) of these 336 subjects who were re-contacted agreed to participate in Phase II and underwent venous blood draw for creatinine determination (Table 3). Table 8 summarizes re-contact and enrollment for Phase II by community.

**Table 7:** Re-contact and enrollment of cohort members in Phase II

Cohort members sought	400
Cohort members re-contacted	336 (84 %)
Agreed to participate in Phase II study	319 (95 %)
Refused to participate in Phase II study	17 (5 %)
Cohort members not re-contacted	64 (16 %)
Died since Phase 1 enrollment	10 (16 %)
Moved (changed residence)	32 (50 %)
Not at home	22 (34 %)

**Table 8:** Summary of participation and enrolment for Phase II. (Note that the municipalities of Altagracia and Myogalpa were new for Phase II, therefore there were no cohort members to re-locate in these two communities)

Municipality	Cohort members sought	Cohort members found and enrolled (phase II)	Deaths	Cohort members not enrolled in Phase II			New participants enrolled
				Moved and not found	Not home	Refused to participate	
Tola	63	56	1	3	1	2	17
Belen	68	55	3	3	4	2	10
Buenos Aires	18	13	0	4	1	0	9
San Juan del Sur	54	49	0	2	3	0	9
San Jorge	46	34	1	4	6	1	22
Potosi	56	39	1	4	5	7	23
Cardenas	33	25	2	4	1	1	12
Rivas	62	48	2	7	1	4	35
Altagracia	NA	NA	NA	NA	NA	NA	42
Moyogalpa	NA	NA	NA	NA	NA	NA	42
Totals	400	319	10	32	22	17	221

There were 10 deaths in these 400 subjects over this 2 year interval (annual mortality rate 1.25 %). The median age of those who died was 68 years and 4 of the 10 deaths occurred in men. Causes of death as reported by household members include “old age”, stroke, kidney disease (2), “lungs”, hypertension (2), myocardial infarction and unknown (2).

### Study expenses

Phase I required 33 days of field work (subject recruitment) and Phase II required 37 days in addition to 8 days required to transport blood samples to Managua. The budgets for Phase I and II were $12,683 and $21,041 respectively excluding travel, room and board for the research assistants which approximated a total of $20,000 for each study phase. Major expenses included vehicle rentals (4 wheel drive SUVs), diesel fuel, driver’s salary and study supplies. Costs for Phase I were reduced by the donation of StatSensor Express testing devices and test strips by Nova Biomedical, Waltham, MA, USA. Weekly transportation and testing of venous blood in Managua added significant cost to Phase II.

## Discussion

The aim of this project was to create a representative geographical-based cohort for the study of chronic kidney disease and other public health issues in the Department of Rivas in Nicaragua and to determine the ability to maintain follow up of the cohort over a two year time period. The cohort was created in 2012 with enrollment of 1021 adults and expanded by 221 subjects in Phase II through additional recruitment from homes visited in Phase I as well as from communities in the two municipalities comprising the island of Ometepe which had not been previously visited (Moyogalpa and Altagracia). Subject enrollment followed a geographical household-based approach with thirty-two communities selected at random within the 10 municipalities. Municipalities were chosen as the geographical units for selection of communities (generally 2–3 communities per municipality) because public healthcare services in Nicaragua are delivered by health centers (Centros de Salud) that are administered by these geographical units. Enrollment of adequate numbers of subjects across all municipalities increases the likelihood of participation of each municipality in future research projects that will use this cohort. This approach does however result in disproportionate representation of municipalities when study enrollment is considered on a per capita basis (see Subjects enrolled per 1000 residents, Table 3). Future planned expansion of the cohort in the more populated municipalities will result in normalization of the number of subjects per 1,000 municipality inhabitants.

The 1242 subjects enrolled in the cohort represent 75 % of adults living in the 533 homes visited. Enrollment of all adults living in a household was possible for 63 % of homes. The refusal rate was 9.3 % (155 adults). The most common reason that 262 (15.5 %) of subjects were not approached to participate is that they were away at work and not available for enrollment during the study team visits. Demographic data on 252 of the 417 non-enrolled subjects indicates that they were disproportionately men who were modestly older and performed work in the agricultural sector (Table 2). Nonetheless, data in Table 5 suggests that the cohort is reasonably representative of the population living in the Department of Rivas.

In Phase II it was possible to identify all homes visited in Phase I despite the lack of home addresses and unmarked and unnamed streets and roads. This was accomplished through use of rough maps, GPS coordinates and photos of homes that were collected in Phase I. Retention of the same local driver from Phase I for Phase II was an important aid. Re-contact was made with 84 % of the 400 subjects that were targeted for enrollment in a follow-up study of CKD. With additional effort and time the study team likely could have re-contacted many of the 54 subjects who were not at home or had moved. The stability of the cohort is not surprising given the duration of time that subjects reported living in their current residence (mean of 20 years) and in the Department of Rivas (mean of 32 years, Table 4).

Household members were extremely welcoming to the study team and were eager to engage in conversation regarding public health issues and the purpose of the project. There was a strong general agreement to participate in future projects as demonstrated by the 95 % participation rate in the CKD follow-up study, despite the need for a venous blood draw, among subjects who were successfully re-contacted.

Demographic, questionnaire (Tables 4 and 6), urinalysis and capillary creatinine results (data not shown) are available for all members of the cohort and blood pressure, height and weight and venous blood creatinine results (data not shown) are available for the 319 subjects who participated in the follow-up CKD project. Researchers interested in accessing the cohort for additional studies are encouraged to contact the corresponding author (LDF).

### Limitations

As discussed previously, because they may have been away at work, men were slightly under-represented in the cohort. The majority of interviews were conducted by native English speakers from the United States and Canada who were fluent in Spanish. Occasional translation difficulties with concepts such as occupational and health history and environmental exposures may have reduced the reliability of data collected on the baseline questionnaire. This cohort is limited to the Department of Rivas and thus may not be representative of other regions in Nicaragua. The cohort was created during two separate visits to the region (2012 and 2014) and although we are unaware of any such changes, there is the possibility that practices changed during that interval that would impact the results of the survey or biological testing.

## Conclusions

There is a scarcity of reliable data for important healthcare problems in Nicaragua. The Rivas Cohort Study constitutes a longitudinal cohort representative of the Department of Rivas with demonstrated good subject retention at two years. All subjects are consented for re-contact and in general enjoy participating in research. It is hoped that the cohort and all collected data will be useful for researchers to conduct additional studies in the region. Photographs and GPS coordinates of homes along with maps of the communities make it feasible to re-contact subjects despite the extremely remote location of many communities. The study team is planning to revisit the communities in the summer of 2017 for additional research activities.

## Additional file

Additional file 1: Orally administered questionnaire. (DOCX 26 kb)
