# Supplementary material for: The Rivas Cohort Study: design and baseline characteristics of a Nicaraguan cohort
Source: BMC Nephrol. 2016 Jul 25;17:93. doi: 10.1186/s12882-016-0320-9 (PMC4959050; doi:10.1186/s12882-016-0320-9)
Supplement: Additional file 1: — Orally administered questionnaire. (DOCX 26 kb) [file 12882_2016_320_MOESM1_ESM.docx]

Additional file 1

**Case Report Form #1:**

**Contact Information and Data Collection Summary**

**Section A. Participant Contact Information**

1^st^ Last Name: _____________________________________

2^nd^ Last Name: _____________________________________

First Name: ______________________________________

Second Name: ____________________________________

Town of Residence: _________________________________

Address: __________________________________________

Cell Phone #: ______________________________________

For how many years have you lived:

1. In the Department of Rivas? ______
2. At the current residence? _____

**Section B. Data Collection Summary**

| **Data Element** | **Collected** | **Not Collected** | **Notes** |
| --- | --- | --- | --- |
| Informed Consent |  |  |  |
| Contact Information (Form #1) |  |  |  |
| Family Contact Form (Form #2) |  |  |  |
| Questionnaire (Form #3) |  |  |  |
| Creatinine Test |  |  |  |
| Urine Dipstick |  |  |  |

Date of Interview: ____________________________

Interviewer Name: _______________________________________

**Case Report Form #2:**

**Family Listing**

**Section A. Immediate Family Members (i.e., those that live in the same residence)**

Please list all family members that live with you in the table below and indicate the best times to contact them for the study.

| **Family Member Name** | **Relationship** | **Best Time to Contact** | **Consented to Participate?** |
| --- | --- | --- | --- |
|  |  |  | Yes / No |
|  |  |  | Yes / No |
|  |  |  | Yes / No |
|  |  |  | Yes / No |
|  |  |  | Yes / No |
|  |  |  | Yes / No |

**Case Report Form #3:**

**Participant Questionnaire and Biologic Data Collection**

**Section A. General**

Do you feel well today?  Yes  No

**Section B. Demographics**

Date of Birth (DD/MM/YYYY): ___ ___ / ___ ___ / ___ ___ ___ ___

(note: if DOB is unknown leave blank and collect approximate age: ___ ___ yrs ___ ___ months

Sex:  Male Female

Race/Ethnicity: _____________________________

**Section C. Biological Tests**

***Finger Stick:***

Run #1 🡪 Serum Creatinine: _________ Measured on Device #: _________

Run #2 🡪 Serum Creatinine: _________ Measured on Device #: _________

***Urine Dipstick:***

Leukocyte Esterase: ___________ Nitrites: _____________ Ketones: _________

Glucose: _______________ Protein: ____________ pH: ________ Sp Gr: ________

Turbidity: Normal / Abnormal

**Section D. Recent Medical Symptoms (past 3 months)**

Over the past three months, have you:

Taken pain medications on more than three days for any reason?

Yes No

Taken antibiotics for any reason?

Yes No

Been diagnosed with a urinary tract infection?

Yes No

Experienced any lower abdominal, back, or flank pain?

Yes No

Experienced any burning or pain during urination?

Yes No

Experienced increased frequency of urination?

Yes No

Experienced any fever or chills?

Yes No

Experienced chistata?

Yes No

Had total-body itching?

Yes No

Have you lost weight unintentionally?

Yes No

Experienced a lot of weakness or fatigue?

Yes No

**Section E. Hydration**

What is the source of the water that you drink in your house? *(more than one box may be checked)*

Well  Bottled

River  Don’t Know

Spring  Other: ________________

Piped

Do you treat your drinking water? *(Boil, add chlorine, etc.)*

Boil  None

Add Chlorine  Other: _________________

Home water filter

What quantity of water do you typically drink each day?

≤ 1 L  2-3 L  4-5 L  6-7 L  8-9 L  ≥ 10 L

How much juice or soda do you drink during a day?

≤ 1 cup  2-3cups  4-5 cups  6-7 cups  8-9 cups  ≥ 10 cups

How much coffee or hot drinks do you drink during a day? ______ cups

How much alcohol do you typically drink each day?

0 drinks  1-2 drinks  3-4 drinks  ≥ 5 drinks

From where do you get the water to drink in your work?

Bring from home  River

Bottled  Piped/Tap

Well  Don’t Know

Spring  Other: __________________

Please indicate how much you agree or disagree with the following statement: “Drinking fluids during a workday is a sign of physical weakness.”

strongly agree  agree  neutral  disagree  strongly disagree

Do you believe that drinking cold water when you are hot could make you sick?

Yes  No

**Section F. Labor Information**

In which industry do you work?

Agriculture  Ranching (animal husbandry)

Fishing  Service (e.g., restaurant, etc)

Transportation  Other: __________________

Business

What is your current occupation? ___________________________

How long have you been at this occupation? __________________________

Is your work done indoors or outdoors? ______________________

Would you consider the work to be manual (e.g., heavy lifting, etc) or not-manual (e.g., desk job)? ____________________________________

How many hours do you work each day? _____________________________

Of the hours that you work each day, how many hours do you think you sweat too much? ____________

How intense do you consider your work (on a scale of 1 to 10)? __________

*1: Not intense at all 10: Very intense*

Do you work for yourself or for an employer?

Self  Employer

What type of products do you cultivate (conditional on #1 above)?

Corn  Peanuts

Beans  Plantains

Sesame  Other: ­­­­­­­­­­­­­­­­­­­­­­­­­­­­­­­­_____________________

In your work do you apply fertilizer, herbicides, insecticides or poison?

Yes  No

If the answer is yes, which fertilizers, herbicides, insecticides, or poisons have you applied in the last 5 years?

_____________________________________________________________________________

_____________________________________________________________________________

_____________________________________________________________________________

What activities do you normally perform in your work?

Activity Months Do you get Do you sweat

per year very tired? a lot?

___________________­­­­­­_______ ________  Y  N  Y  N

___________________­­­­­­_______ ________  Y  N  Y  N

___________________­­­­­­_______ ________  Y  N  Y  N

___________________­­­­­­_______ ________  Y  N  Y  N

Does your work require any heavy lifting or repetitive motions?

Yes  No

**Labor History**

Did you have another job before your current one?

Yes  No

How old were you when you started your first job? ___________

Have you worked with sugarcane?

Yes  No

Have you worked with mixing poisons or insecticides?

Yes  No

Have you worked with poison or pesticide application?

Yes  No

Have you worked bagging bananas/plantains in treated bags?

Yes  No

| **Section G. Medical History** |
| --- |

Have you suffered or do you now suffer from any of these illnesses?

High blood pressure  Yes  No  Don’t know

High blood sugar or diabetes  Yes  No  Don’t know

Kidney Disease  Yes  No  Don’t know

Kidney Stones  Yes  No  Don’t know

Has your father, mother, or siblings suffered from: *(Mark only those that apply to subjects’ father, mother, or sibling and specify relationship on the line.)*

High blood pressure  Yes: _______________  No  Don’t know

High blood sugar or diabetes  Yes: _______________  No  Don’t know

Kidney Disease  Yes: _______________  No  Don’t know

Kidney Stones  Yes: _______________  No  Don’t know

Do you smoke?

No, I have never smoked

Yes, I am a current smoker

I used to smoke but don’t anymore

Where do you get your medical care? ___________________________

Observations (Note whether the information is reliable, and if there is anything relevant that the subject said that was not asked specifically on the questionnaire)

___________________________________________________________________________

____________________________________________________________________________

____________________________________________________________________________

____________________________________________________________________________

____________________________________________________________________________
